# Effect of exercise therapy on adolescent idiopathic scoliosis in mild to moderate: a systematic review and network meta-analysis

**DOI:** 10.3389/fmed.2025.1708970

**Published:** 2025-11-20

**Authors:** Hongfang Zhu, Chengjun Li, Zhibo Tian, Qiang Zhang

**Affiliations:** 1 School of Physical Education, Shaanxi Normal University, Xi'an, China; 2 College of Physical Education and Health, Guangxi Normal University, Guilin, China

**Keywords:** idiopathic scoliosis, exercise, adolescent, network meta-analysis, exercise intervention

## Abstract

**Objectives:**

This study aimed to identify the most effective exercise for reducing the Cobb angle and the angle of trunk rotation (ATR) and improving the quality of life (QoL) in adolescents with idiopathic scoliosis (AIS).

**Methods:**

A systematic search of EMBASE, Web of Science, PubMed, Cochrane Library, and MEDLINE identified randomized controlled trials (RCTs) on exercise interventions for light to moderate AIS. Eligible studies involved AIS patients receiving exercise therapy, with outcomes measured by the Cobb angle, ATR, and quality of life scores. The search included articles published from the inception of these databases until June 2025. Two independent researchers conducted screening, data extraction, and quality assessment using the Cochrane risk of bias tool. Network meta-analysis (NMA) was performed using Stata 16.0 and reported per Preferred Reporting Items for Systematic Reviews and Meta-Analyses (PRISMA)-NMA guidelines. The protocol was registered in the International Prospective Register of Systematic Review (PROSPERO) (CRD42024557874).

**Results:**

A total of 16 RCTs involving 600 participants were included in the study. Compared to controls, Schroth exercise significantly improved the Cobb angle [SMD = -1.42, 95% CI (−2.03, −0.80)] and ATR [SMD = -1.86, 95% CI (−3.05, −0.68)]. The Lyon method showed the most significant improvement in quality of life [SMD = 2.64, 95% CI (0.80, 4.49)]. The Scientific Exercises Approach to Scoliosis (SEAS) exercise also helps improve the Cobb angle, but its effect is weaker.

**Conclusion:**

Exercise therapy effectively reduces spinal curvature and enhances the quality of life in adolescents with AIS. Schroth exercises are most effective for improving the Cobb angle and ATR, while the Lyon therapy significantly benefits quality of life. However, due to small sample sizes, substantial between-study heterogeneity, and risk of bias, the overall certainty of evidence is low; the results should be interpreted cautiously.

**Systematic review registration:**

PROSPERO (CRD42024557874), https://www.crd.york.ac.uk/PROSPERO/view/CRD42024557874.

## 1 Introduction

Adolescent idiopathic scoliosis (AIS) is a three-dimensional structural alteration in the form and location of the spine column, thorax, and trunk (1), which is diagnosed by a frontal plane scoliosis angle (Cobb’s angle) of >10°accompanied by axial rotation. The prevalence of AIS ranges from 0.47 to 5.2% (2), and its incidence appears to be increasing (3). During childhood and adolescence, the rapid growth of the skeletal and muscular systems and rise in poor posture may accelerate the progression of scoliosis, leading to generalized pain, impaired respiratory and cardiovascular function, and adverse effects on mental health that collectively impair quality of life (4).

Scoliosis treatment is determined based on the patient’s specific form of scoliosis, age, curvature size, and progression risk, with surgical and conservative treatments being the main options (5, 6). For patients with mild to moderate scoliosis, brace treatment has achieved good therapeutic results (7). For low Cobb angles, nighttime brace wear minimizes disruption to daily life. However, curves exceeding 35° typically require full-time support (often 18–23 h/day) to limit progression (8). This heavy burden may lead to functional deterioration, including muscle stiffness and weakness, which can hinder rehabilitation (9). In recent years, exercise interventions have been recognized as an easy-to-use and highly effective conservative treatment frequently suggested to reduce progression; rectify postural behavior; enhance the neuromotor function of the spine; and improve flexibility, muscle strength, and elasticity of the spine and thorax (10). Combined with braces, exercise interventions as an adjunct to orthotics can reduce the impairment of respiratory function caused by orthotics and enhance the patient’s physical abilities (11).

Although the emergence of high-quality trials in AIS exercise interventions has led to evidence syntheses, these are controversial. First, many studies compare physiotherapeutic scoliosis-specific exercises (PSSE) only with generic exercise programs or no treatment, which precludes estimating the efficacy of individual exercise schools (12, 13). Second, numerous reports either focus on a single school or include brace + exercise co-interventions, making it difficult to isolate the effect of exercise alone (14, 15). These limitations obscure head-to-head comparisons across exercise schools. Based on this, this study used a reticulated meta-analysis comparing commonly used exercise regimens for AIS, evaluating Cobb angle, ATR, and quality of life to inform clinical practice. We hypothesized that PSSE, particularly Schroth, would yield superior outcomes compared with generic exercise or standard care.

## 2 Materials and methods

This systematic review and network meta-analysis (NMA) follows the principles specified in the PRISMA statement. The study protocol was formally registered with the International Prospective Register of Systematic Reviews (PROSPERO) and assigned the registration number CRD42024557874.

### 2.1 Search strategy

Two researchers (Q. Z. and H. Z.) independently searched the EMBASE, Web of Science, PubMed, Cochrane Library, and MEDLINE databases from inception to June 2025. Search terms used included “scoliosis,” “AIS,” “exercise,” and “clinical trials.” To avoid literature omissions, studies included in this review and the references of research included in previous systematic reviews and meta-analyses were manually checked to identify any additional pertinent studies (See Supplementary Table S3 for specific search strategies).

### 2.2 Scope

This NMA included studies investigating the efficacy of various exercise therapies for adolescent idiopathic scoliosis (AIS). The specific inclusion criteria were: (1) participation criteria: individuals with a confirmed diagnosis of AIS in mild to moderate; (2) intervention type: any exercise intervention, defined as a planned, structured, and repetitive physical activity, regardless of how often, how long, or how vigorous; (3) type of comparison: direct comparisons between the exercise therapies retrieved by the main or comparisons with usual care/no exercise are not limited by the frequency and duration of interventions; (4) outcome indicators: ① Cobb angle, ② ATR (angle of trunk rotation), and ③ SRS-22; and (5) the design of the included studies was a randomized controlled trial (RCTs).

The criteria for exclusion were: (1) repeated publications (multiple submissions and academic misconduct); (2) studies that integrate exercise with additional health strategies (for example, combining bracing with exercise therapy); (3) studies of reviews, case reports, conference papers, and literature for which raw data are not available and for which data cannot be extracted; and (4) reports written in languages other than English (Only English-language full texts were included to ensure consistent risk of bias appraisal, standardized intervention and outcome coding, and comparability of reporting).

### 2.3 Study selection and data extraction

EndNote X9 software was used to eliminate duplicate literature during the study selection process. Two coauthors (H. Z. and C. L.) independently evaluated the titles and abstracts of the retrieved records using a double-blind methodology, adhering to the established inclusion and exclusion criteria. Full texts of studies that potentially met the inclusion criteria were downloaded for further screening. Before formal screening, we conducted a calibration exercise on a sample set to harmonize judgments and refine decision rules. In cases of disagreement between the two coauthors, a third coauthor (Z. T.) participated in a joint discussion to determine whether to include the study.

Two coauthors (Q. Z. and H. Z.) autonomously extracted the subsequent data from every included article using a pre-designed form throughout the data extraction process. The following information will be examined: (1) fundamental study information: first author, year of publication, and country; (2) the characteristics of the participant details: sample size, age, and disease duration; (3) characteristics of the intervention parameters: intervention time, frequency, kind, and training regime; and (4) baseline and endpoint outcome data.

### 2.4 Risk of bias assessment

Two researchers (H. Z. and L. W.) independently assessed the risk of bias in the randomized controlled trials (RCTs) included in the study using the Cochrane Collaboration’s risk of bias tool. Any dispute was resolved through agreement or deliberation with an additional reviewer (Q. W.). This instrument assesses the potential for bias based on seven fields: (1) the randomized procedure, (2) the hidden distribution, (3) the blinding of participants and personnel, (4) the blinding of outcome assessment, (5) incomplete outcome data, (6) selective reporting, and (7) other sources of bias.

### 2.5 Data synthesis and statistical analysis

This study used standardized mean difference (SMD) and 95% confidence intervals (CI) as effect sizes. To avoid any potential washout effect, the conclusion of the therapy was consistently defined as the termination of exercise involvement. The change in standard deviation was converted using the formula provided in the Cochrane Handbook (version 6.5) (16). The quality of the literature was assessed, and the risk of bias was plotted using RevMan 5.4 software. A reticulated meta-analysis was conducted using Stata 16, which is based on a frequency-based framework (17). A random-effects model was used to merge effect sizes.

NMA analysis was performed following the PRISMA-NMA statement. The initial step involved visualizing the network structure using a network geometry plot. In this graph, the area of each node corresponds to the number of trials encompassed by every instance of involvement, and the thickness of the interconnecting lines between nodes is proportional to the number of trials directly compared to the two interventions. Secondly, consistency was tested using Wald tests and node splitting methods to determine whether treatment effects derived from direct comparisons were consistent with those derived from indirect comparisons (See Supplementary Table S4). Any significant inconsistency would suggest a potential violation of transitivity assumptions and reduced confidence in network estimates. Standard pairwise meta-analyses were then conducted for all direct comparisons using the random-effects DerSimonian–Laird model (18). Between-study heterogeneity for each pairwise comparison was quantified using the I^2^ statistic. Between-study heterogeneity was assessed to determine whether the observed variation in treatment effects could be attributed to actual differences among studies rather than random error. Finally, a forest plot was created to present these findings visually. Three authors (H. Z., C. L., and Q. X.) assessed transitivity by evaluating whether the direct comparisons of interventions were conducted in study samples with comparable baseline clinical characteristics. It was assumed that the populations in these studies shared similar baseline distributions of key effect modifiers, such as age, disease severity, and disease duration. To assess the robustness of the estimates and determine whether specific studies accounted for most of the heterogeneity, we conducted a sensitivity analysis that excluded studies with a high risk of bias (See Supplementary Figure S3).

To identify publication bias or small sample effect, a funnel plot and Begg’s test were used. A *p*-value of < 0.05 was considered significant (see Supplementary Figures S4, S5). The ranking of exercise interventions was determined by comparing the relative ranks of different treatments. These rankings were then visually represented using rankograms, and the surface under the cumulative ranking (SUCRA) was computed for every exercise. The SUCRA method assigns an arbitrary value ranging from 0 to 1 to facilitate their classification in the rankogram. An optimal intervention would achieve an SUCRA value approaching 1, while a poor intervention would have a value close to 0.

### 2.6 The overall strength of the evidence

Two authors (H. Z. and Q. W.) evaluated the grading of evidence for the outcomes according to the GRADEpro Guideline Development Tool (19). The evaluations were categorized into risk of bias, inconsistency, indirectness, imprecision, and publication bias. Each domain was classified as “not serious,” “serious,” or “very serious” according to the evaluation criteria, and the overall certainty of the evidence was categorized into four grades: very low, low, moderate, or high. (Table 1).

**Table 1:** Grade evidence level quality assessment.

Outcome	No. of studies	Study design	Risk of bias	Inconsistency	Indirectness	Imprecision	Other considerations	Overall certainty
Cobb angle	16	Randomized trials	Not serious^a^	Serious^b^	Serious^c^	Not serious	None	Low ^a,b,c^
ATR	10	Randomized trials	Not serious^a^	Serious^b^	Serious^c^	Not serious	None	Low ^a,b,c^
SRS-22	7	Randomized trials	Not serious^a^	Serious^b^	Serious^c^	Not serious	None	Low ^b,c^

^a^Some studies did not report random allocation methods and blinding. ^b^Patient inclusion criteria are within range but not completely consistent. ^c^Comparing the effectiveness of different intervention methods in research relies on indirect comparison.

## 3 Results

### 3.1 Study selection

**Figure 1:**
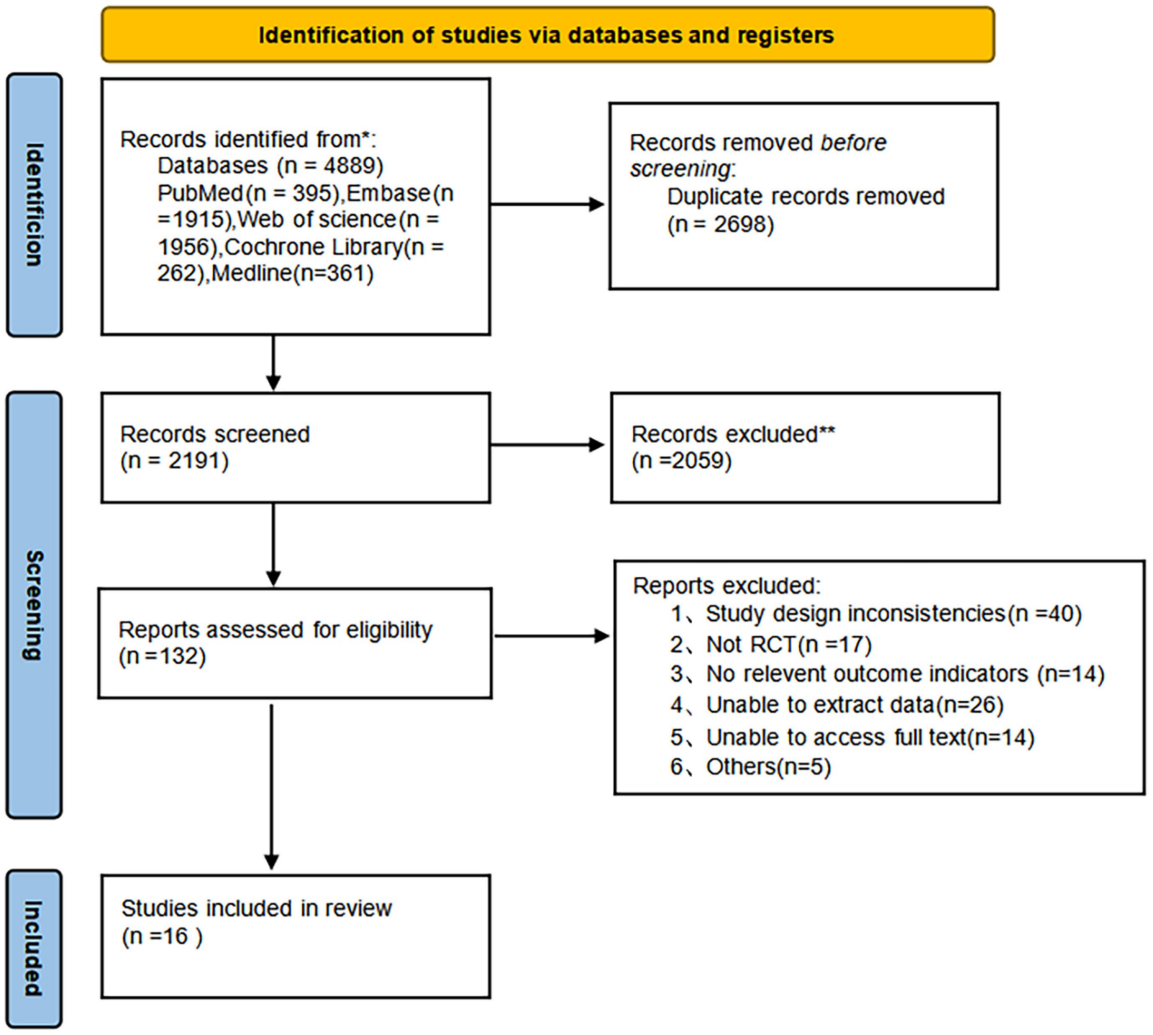
Literature screening process consistent with the study. Created from our own data using the official PRISMA 2020 template (CC BY 4.0).

A total of 4,889 full-text articles were discovered, including Web of Science (*n =* 1956), PubMed (*n =* 395), Embase (*n =* 1915), Cochrane Library (*n =* 262), and MEDLINE (*n =* 361). Overall, 132 studies advanced to the stage of undergoing a comprehensive assessment of the complete manuscript. Of these, 116 studies were eliminated because they failed to match our requirements for inclusion. A total of 16 studies were included in the current review—a comprehensive depiction of the step-by-step procedure in Figure 1.

### 3.2 Characteristics of the included studies

Table 2 displays the characteristics of the studies included. From the included trials, the year of study publication was concentrated in the last 10 years. A total of 600 patients from 8 countries were enrolled, of which 313 cases were in the group experimenting and 287 cases in the corresponding control group. The exercise therapies include 9 articles on Schroth, 1 on Lyon, 3 on SEAS, 4 on core stability exercises (CS), 3 on Pilates, and 2 on proprioceptive neuromuscular facilitation (PNF). A total of 16 studies were reported on the outcome indicator Cobb, 10 on the ATR, and 7 on the SRS-22 scale.

**Table 2:** Characteristics of the included studies.

Study	Country	Sample(n)		Mean age(T/C)	Cobb	Frequency and intensity	Duration (month)	Exercise		Outcome indicator
		T	C					T	C	
Büyükturan et al. (30)	Turkey	15	16	14 ± 1.9/14.2 ± 2	10–30	3 days a week, 90 min/day Initial intensities: 3–5 sets of 4–6 repetitions each Target intensities: 3–5 sets of 6–10 repetitions each	6	Schroth	Lyon	①②③
El et al. (40)	Egypt	15	15	11.5 ± 1.8/12.03 ± 1.94	10–20	3 days a week, 60 min/day The exercises consisted of warm-up (10 min), the main exercise (40 min), and cool-down (10 min)	3	CS	SEAS	①③
Khaledi et al. (41)	Iran	15	10	16.27 ± 1.44/15.4 ± 1.51	10–20	3 days a week, 50–70 min/day The exercises consisted of a warm-up (10 min), the main exercise (40–55 min), and cool-down (5 min) 4–6 sets × 5–8 repetitions	3	Schroth	C	①②③
Ko and Kang (42)	Korea	15	14	12.71 ± 0.72/12.8 ± 0.86	10–20	3 days a week, 60 min/day Warming up (10 min), followed by exercises (40 min), and terminated with cooling down (10 min) 3 sets × 12 repetitions The rating of perceived exertion was used to set the exercise intensity to somewhat difficult	3	CS	C	①
Manzak Dursun et al. (43)	Turkey	16	15	10–18	25–50	7 days a week, 60 min/day Warming up (10 min), followed by exercises (40 min), and terminated with cooling down (10 min) 12 repetitions/session	3	Pilates	C	①②
Kuru et al. (44)	Turkey	15	15	12.9 ± 1.4/12.8 ± 1.2	20–50	3 days a week, 90 min/day	6	Schroth	C	①②③
Monticone et al. (27)	Italy	55	55	12.5 ± 1.1/12.4 ± 1.1	10–25	1 day a week and 2 days a week at home 60 min at outpatient 30 min at home	6	SEAS	C	①②③
Kocaman et al. (10)	Turkey	14	14	14.07 ± 2.37/14.21 ± 2.19	10–25	3 days a week, 90 min/day The exercises consisted of warm-up (10 min), the main exercise (70 min), and cool-down (10 min) Initial intensities: 7–10 repetitions each Target intensities: 10–15 repetitions each	2.5	Schroth	CS	①②③
Hwangbo (45)	Korea	8	8	18.14 ± 1.6/18.88 ± 1.55	≥20	3 days a week, 60 min/day The exercises consisted of warm-up (10 min), the main exercise (45 min), and cool-down (5 min)	3	Schroth	Pilates	①
Mohamed and Yousef (46)	Egypt	17	17	14.5 ± 1.2/14.9 ± 1.4	10–25	3 days a week, 60 min/day E: 4 sets of 6 repetitions C: 2 sets of 10 repetitions	6	Schroth	PNF	①②
Won et al. (47)	Korea	10	10	14.5 ± 2.5/15.9 ± 2.69	10–20	3 days a week, 30 min/day Four steps: twenty repetitions with a 10-s hold	6	PNF	C	①
Kim and HwangBo (48)	Korea	12	12	15.3 ± 0.8/15.6 ± 1.1	≥20	3 days a week, 60 min/day E: The exercises consisted of warm-up (10 min), stretching (stretching the chest part: 5 min), the main exercise (40 min), and cool-down (5 min). C: The exercises consisted of warm-up(10 min), the main exercise(45 min), and cool-down (5 min)	3	Schroth	Pilates	①
Gao et al. (49)	China	43	21	10–18	20–40	2–3 days a week, 60 min/day	24	Schroth	C	①③
Akyurek et al. (50)	Turkey	15	14	13.73 ± 1.83/13.86 ± 1.86	10–30	2 days a week, 45–60 min/day	2	Schroth	C	①②
Kisa et al. (51)	Germany	25	25	11.76 ± 2.26/11.4 ± 2.73	10–45	2 days a week Three sets of five repetitions	6	Schroth	CS	①②
Yuan et al. (52)	China	23	26	4–8	10–20	5 days a week, 45–60 min/day	12	SEAS	C	①②

This table summarizes the core features of the included RCTs evaluating exercise-based interventions for AIS. “T” refers to the treatment group; “C” refers to the control group. Age is reported as mean ± standard deviation. Exercise frequency, intensity, and session structure are described in detail. Exercise types include Schroth therapy, core stabilization (CS), Scientific Exercise Approach to Scoliosis (SEAS), Pilates, proprioceptive neuromuscular facilitation (PNF), and conventional therapy (C). Outcome indicators are coded as follows: ① Cobb angle; ② trunk rotation; and ③ quality of life.

### 3.3 Risk of bias assessment, publication bias, and inconsistency

**Figure 2:**
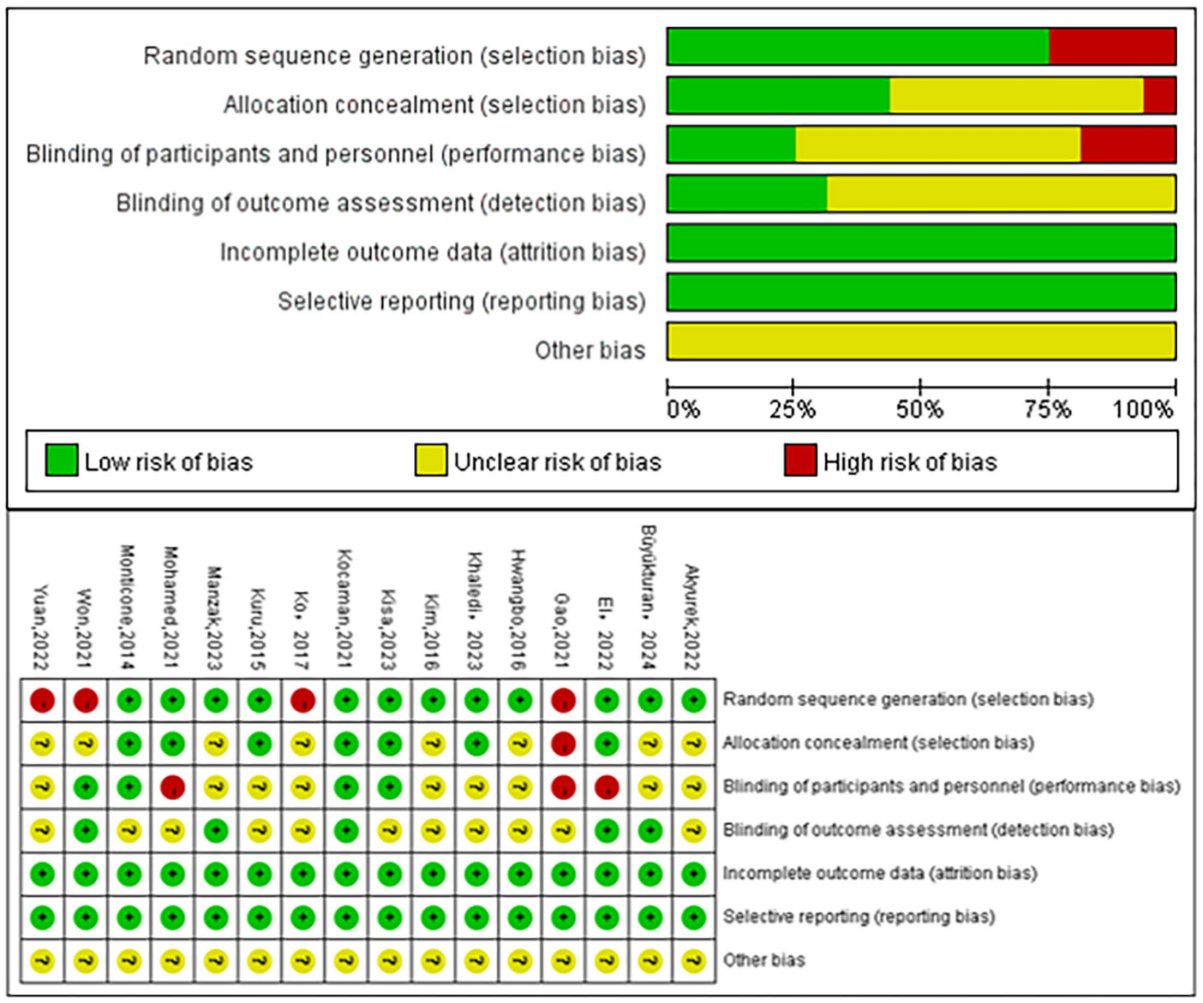
Literature quality assessment chart. Each domain is evaluated for all included studies. Risk levels are color-coded as follows: green = low risk, yellow = unclear risk, red = high risk.

The results of the quality evaluation for 16 articles are illustrated in Figure 2. Among these, 13 articles detail the specific methods of randomization, 7 articles report allocation concealment, 4 articles implement participant blinding, 5 articles utilize assessor blinding, 16 articles maintain data integrity, and 16 articles provide selective reporting. The highest risk of bias arises from the lack of participant blinding. Many studies show insufficient information on the other risk of bias indicators. (Supplementary Table S2). Consequently, the true extent of bias in the included studies remains uncertain. Three funnel plots depict the publication bias of the included studies for each of the three metrics (Supplementary Figure S3), demonstrating overall symmetry of each metric. Begg’s test was performed separately for articles included for each indicator, and the results were all *p* > 0.05 (Supplementary Table S4). No substantial publication bias was seen among the articles included in the network meta-analysis. The network meta-analysis revealed no discrepancy regarding global inconsistency (Supplementary Table S4).

### 3.4 Network meta-analysis

**Figure 3:**
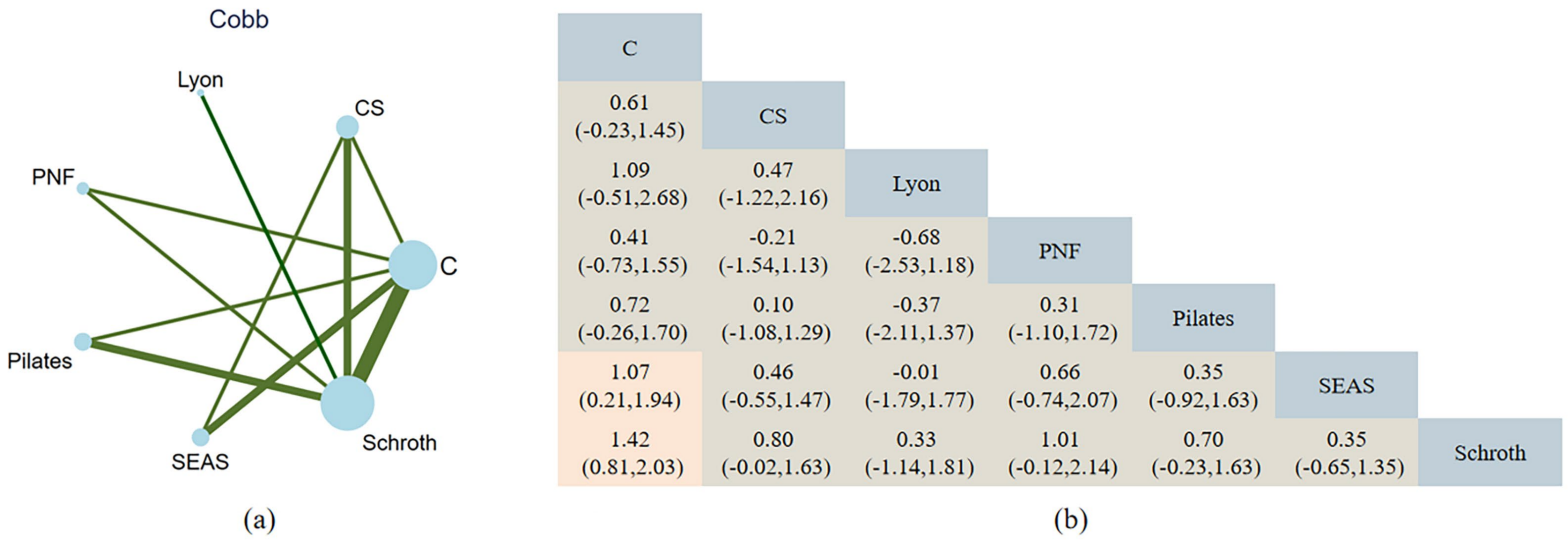
**(a)** Network plot of exercise interventions for Cobb angle. Node size reflects the number of studies; line thickness indicates the number of direct comparisons. **(b)** Pairwise network meta-analysis results (SMDs with 95% CIs). Negative values favor the row intervention over the column comparator.

**Figure 4:**
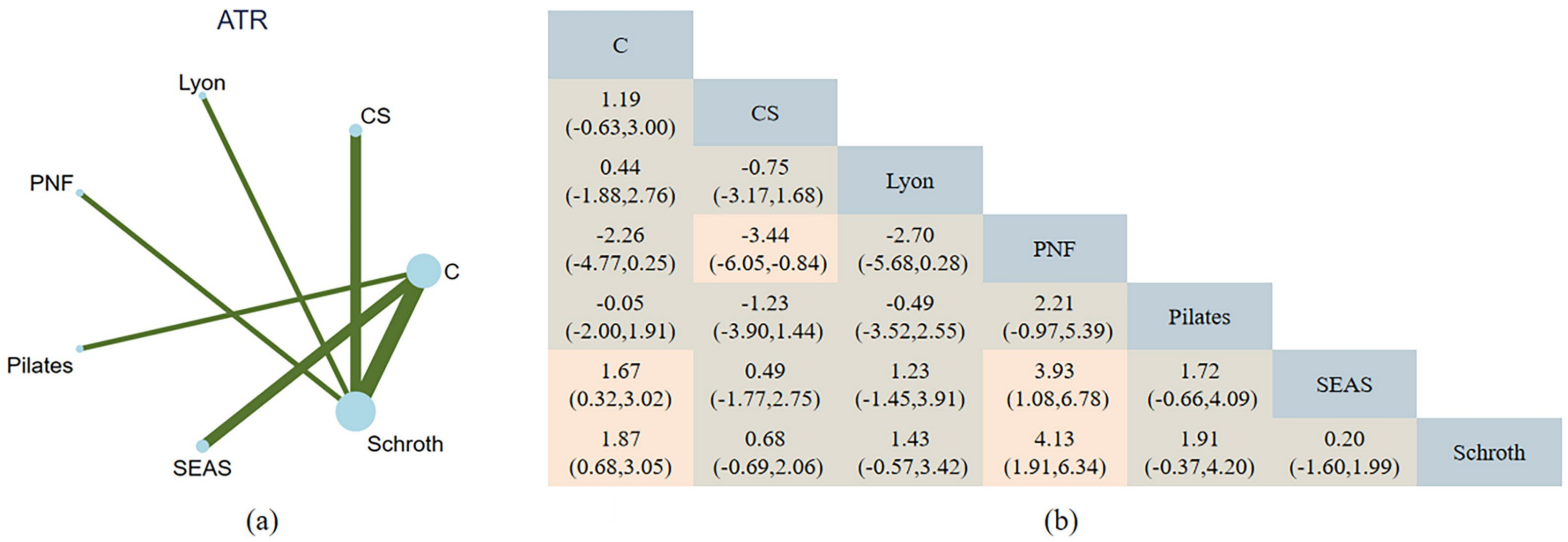
**(a)** Network plot of exercise interventions for ATR. Node size reflects the number of studies; line thickness indicates the number of direct comparisons. **(b)** Pairwise network meta-analysis results (SMDs with 95% CIs). Negative values favor the row intervention over the column comparator.

**Figure 5:**
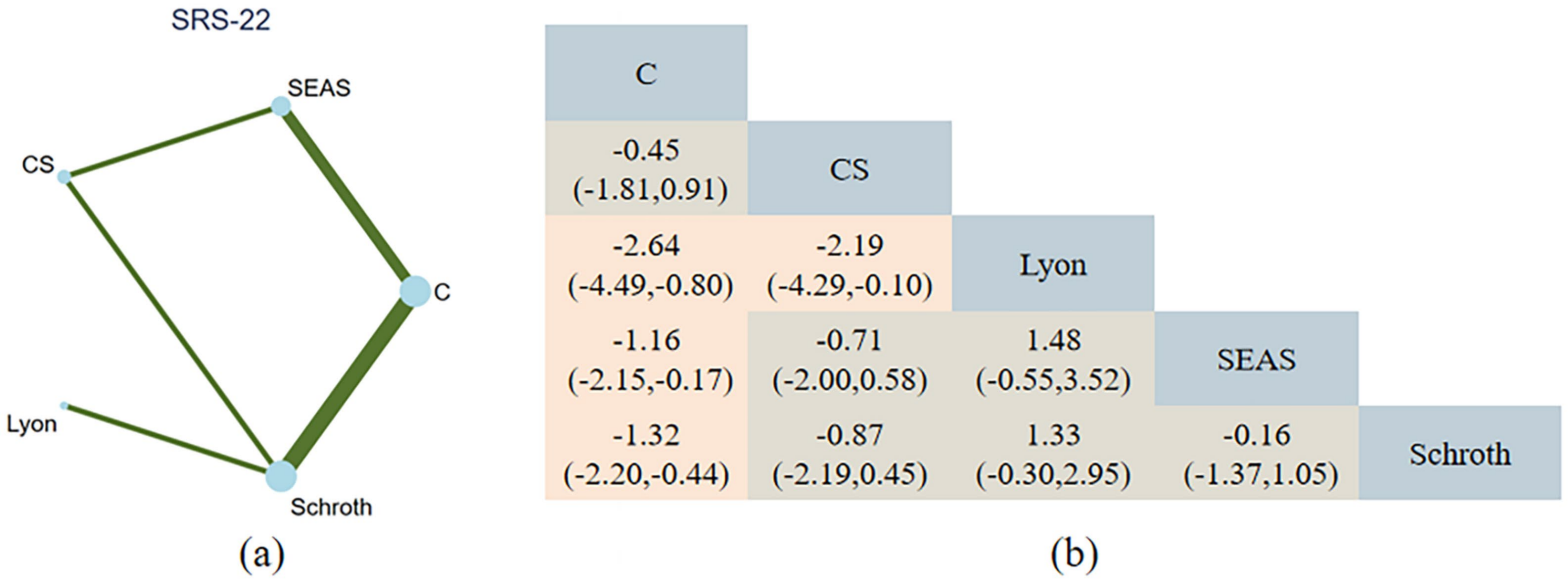
**(a)** Network plot of exercise interventions for SRS-22. Node size reflects the number of studies; line thickness indicates the number of direct comparisons. **(b)** Pairwise network meta-analysis results (SMDs with 95% CIs). Positive values favor the row intervention over the column comparator.

**Figure 6:**
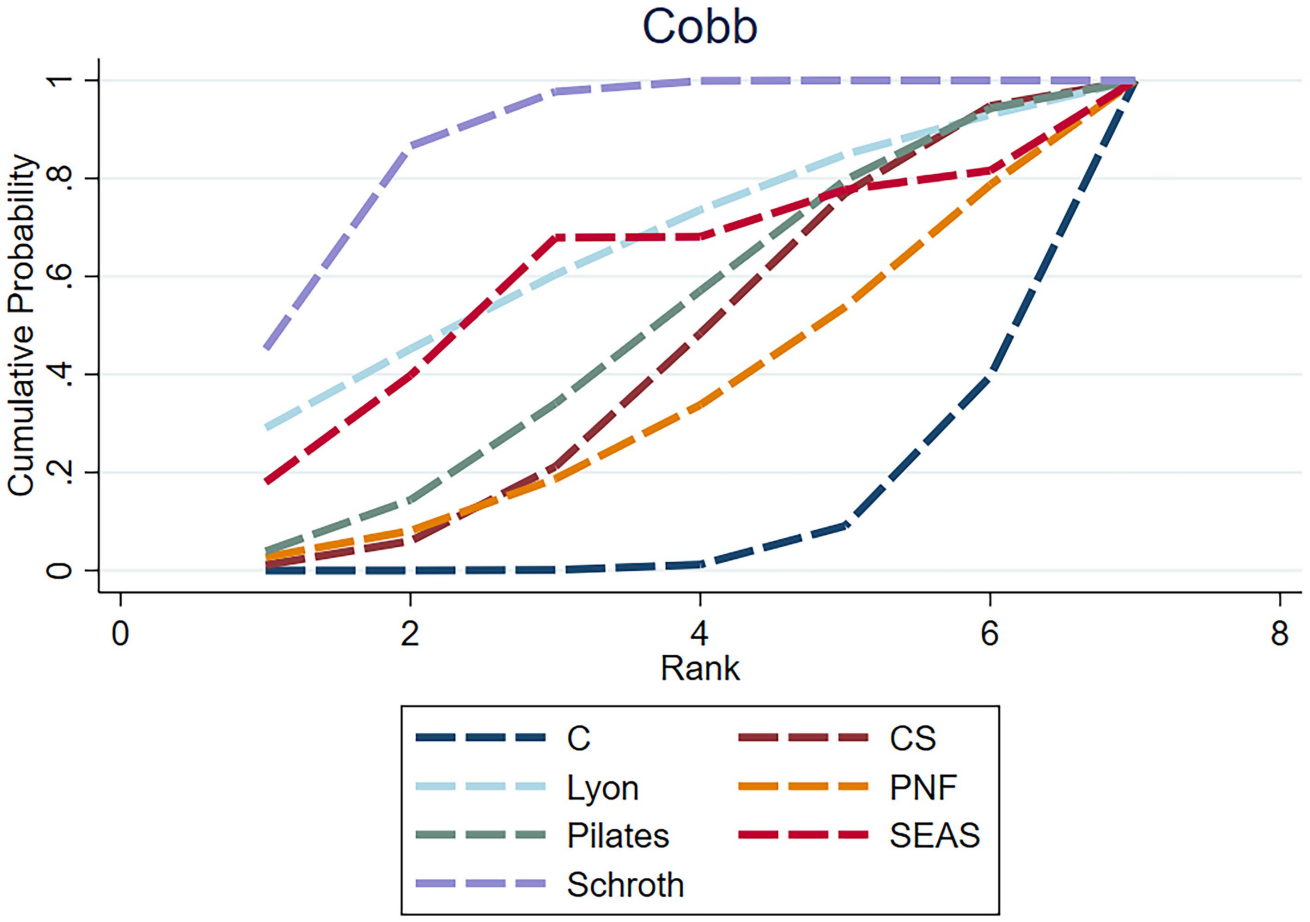
Rankogram for each intervention affecting the Cobb angle in AIS. The curves represent the probability of each intervention being ranked at each position. Higher left-shifted curves indicate better ranking.

**Figure 7:**
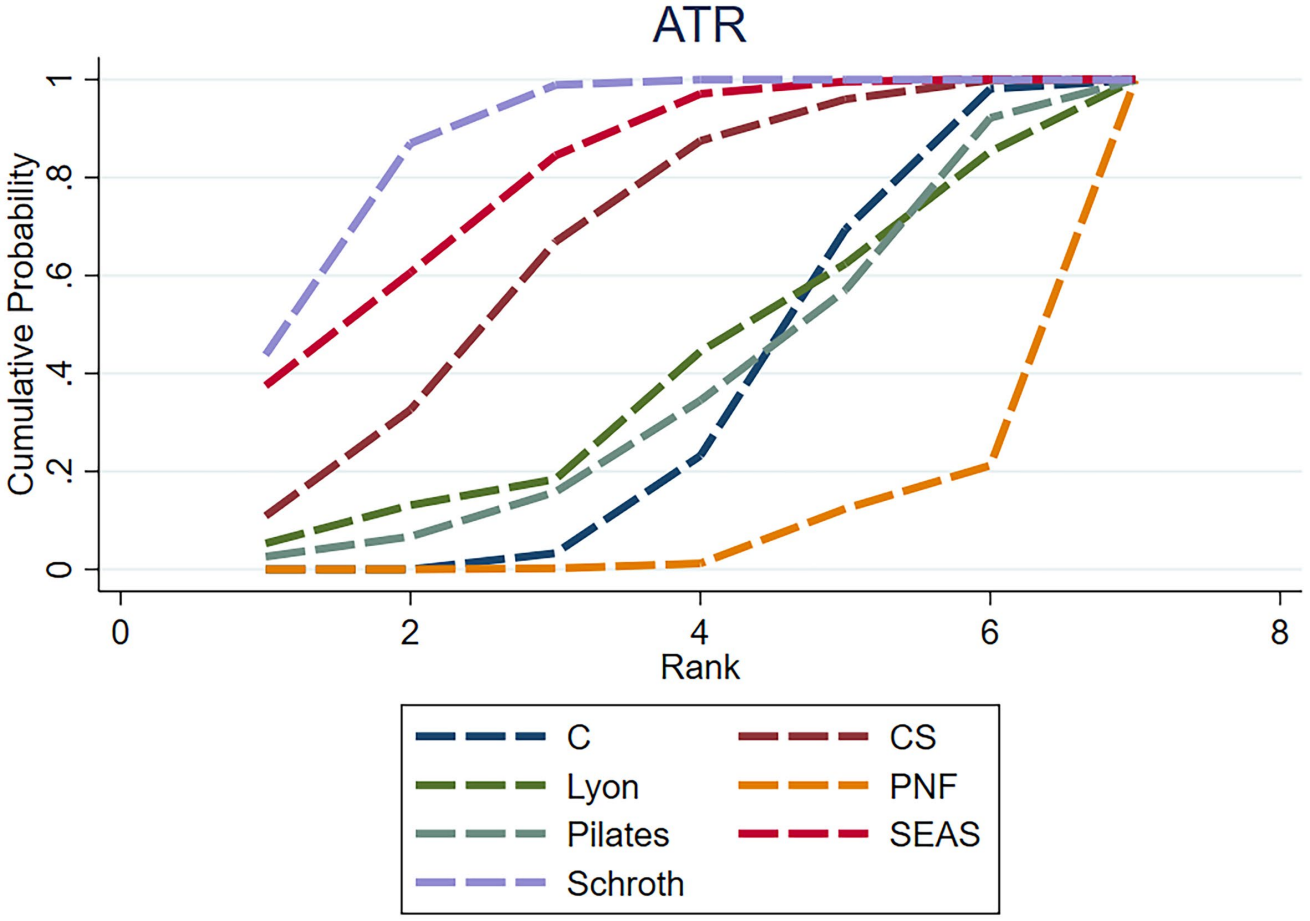
Rankogram for each intervention affecting the ATR in AIS. The curves represent the probability of each intervention being ranked at each position. Higher left-shifted curves indicate better ranking.

**Figure 8:**
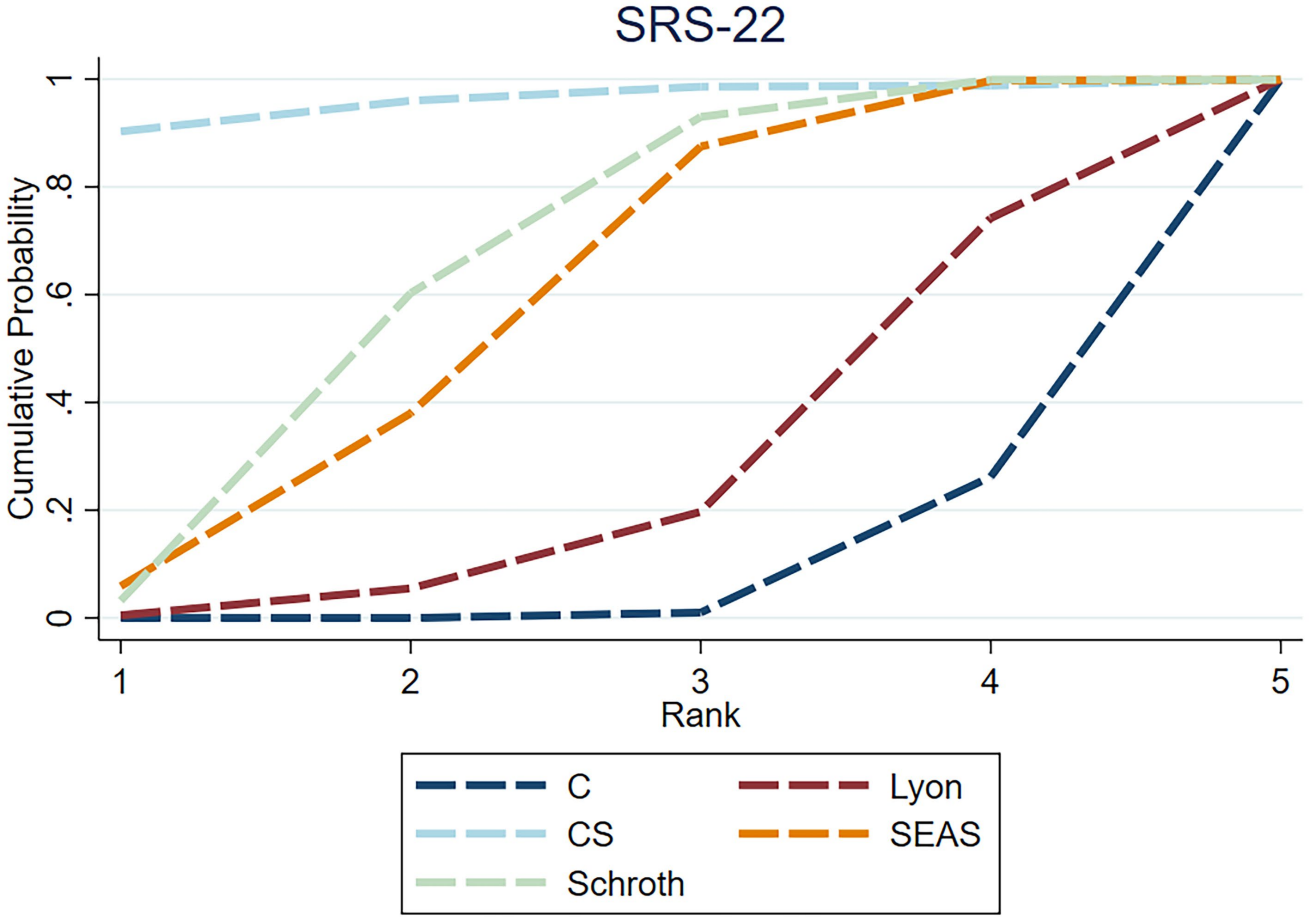
Rankogram for each intervention affecting the SRS-22 in AIS. The curves represent the probability of each intervention being ranked at each position. Higher left-shifted curves indicate better ranking.

The NMA includes three indicators on patients with AIS: Cobb, ATR, and SRS-22. All networks adhere to the criteria of consistency, heterogeneity, and transitivity. Figures 3–5 show NMA maps and a comprehensive results matrix from research examining the efficacy of exercise on Cobb angle, ATR, and SRS-22 scores. The rankings are shown in Figures 6–8, respectively.

The NMA assessment was performed using data from 16 studies, which included a total of 600 people and six intervention categories. Schroth [−1.42, 95%CI (−2.03, −0.80)] and SEAS [−1.07, 95%CI (−1.94, −0.21)] significantly reduced the Cobb angle in patients with AIS, consistently outperforming the control group. Other comparisons between different exercise therapy groups in improving the Cobb angle were insignificant (*p* > 0.05). Figure 6 shows the ranking of exercise therapies according to their potential to have the greatest or least impact on the Cobb angle. The exercise intervention with the highest potential for reducing the Cobb angle (SUCRA value) is Schroth (SUCRA = 88.8). The strength of the certainty was very low. Begg’s test for publication bias was not statistically significant (*p* > 0.05).

The NMA assessment was conducted based on ten studies, which involved 417 people and covered 6 intervention categories. For ATR, Schroth [−1.86, 95% CI (−3.05, −0.68)] and SEAS [−1.67, 95% CI (−3.02, −0.68)] were determined to be much more effective than the control in minimizing ATR. Although some comparisons were not significant among the different interventions, PNF demonstrated poorer outcomes. Schroth is the best intervention in online comparisons for reducing ATR (SUCRA = 88.8) (Figure 7). The strength of the certainty was low. Begg’s test for publication bias was not statistically significant (*p* > 0.05).

The NMA for SRS-22 contained seven studies, with 318 individuals. A total of four of the six exercise intervention categories were represented in these investigations. Compared with the control group, Lyon [2.64, 95%CI (0.80, 4.49)], Schroth [1.32, 95%CI (0.44, 2.20)], and SEAS therapy [1.16, 95%CI (0.17, 2.15)] were determined to exhibit significantly greater efficacy than the control in enhancing the quality of life, and Lyon therapy was superior to CS therapy [2.19, 95%CI (0.10, 4.29)]. Despite limited evidence, Lyon (SUCRA = 96.2) is the most effective exercise intervention for improving the quality of life (Figure 8). The strength of the certainty was very low. Begg’s test for publication bias was not statistically significant (*p* > 0.05).

## 4 Discussion

In clinical practice, a variety of exercise modalities are widely used in the treatment of AIS. Our NMA synthesized direct and indirect evidence from 16 RCTs that included 600 adolescents and compared six exercise therapies on Cobb angle, ATR, and SRS-22 scores in patients with AIS. Our primary findings indicate that the Schroth exercise intervention shows the most promise for reducing Cob and ATR, followed by the SEAS. At the same time, Lyon demonstrates excellent effectiveness in improving QoL. Among the six interventions examined, PNF was deemed the least effective. The SUCRA ranking reveals that when selecting interventions to improve the condition of AIS patients, Schroth and SEAS rank as the top two exercise interventions for this population.

Patients with AIS often present with uneven shoulders and asymmetrical waist circumference (20). The etiology of this multifaceted and intricate symptom remains poorly delineated, and multiple causes could likely contribute to its development. Given the growing body of research on exercise interventions for patients with AIS, Schroth has received increasing attention for its benefits on the Cobb angle. Our results suggested that Schroth was significantly more effective in improving the Cobb angle and ATR in patients with AIS than the control group, including standard care and no intervention. These findings are consistent with results reported in previous meta-analyses examining the effects of Schroth exercises on adolescent idiopathic scoliosis that showed reductions in vertebral angulation and trunk asymmetry following the implementation of corrective, therapeutic Schroth exercises (21, 22). Furthermore, a meta-analysis by Wang et al. (23) indicates that while the Schroth method significantly improves Cobb angles and ATR, the SEAS method is also listed as the most effective intervention for reducing Cobb angles. We similarly observed SEAS as beneficial for AIS, second only to Schroth. However, the study by Seleviciene et al. (24) demonstrated that among PSSE modalities, only the Schroth method significantly reduced ATR. This finding clearly contradicts the results of the meta-analysis conducted in this study. These discrepancies likely reflect differences in study mix and intervention definitions (inclusion of combined programs vs. single-modality analyses). A recent network meta-analysis identified yoga as the most effective intervention for adolescent idiopathic scoliosis (12); this finding is inconsistent with our results. The discrepancy may broadly reflect methodological limitations in that analysis. Specifically, the study misclassified specific exercise modalities, inconsistently grouping physiotherapeutic scoliosis-specific exercises (PSSE). In addition, the number of included trials was limited, reducing the representativeness and robustness. Concerning the impact of exercise on the QoL in individuals diagnosed with scoliosis, several studies have documented the beneficial effect of various exercise programs on QoL (25–27). In contrast, certain studies failed to see any effect of exercise on enhancing the QoL in patients with AIS. Nevertheless, the lack of consistency among these studies makes it difficult to determine the impact of various workouts on the QoL of patients. The findings of this study substantiate that exercise enhances the QoL. Compared with conservative treatment, the Lyon, Schroth, and SEAS effectively improve QoL of AIS patients, with the Lyon showing the most pronounced effect. However, previous studies have indicated that only the Schroth or Schroth therapy combined with core muscle training can significantly improve quality of life (28). In our network, the number of Lyon trials was small, making its node sparse and rankings more sensitive to single studies, thereby limiting. Therefore, further large-scale RCTs are also needed to clarify exercise modalities’ comparative effects on structural and psychosocial outcomes in AIS. The Lyon method is a phased program that incorporates thoracic flexion to create a coupled movement of derotation and opening of the concavity, alongside axial elongation and segmental stabilization. Progression adds core work (often on a Swiss ball) with visual/vestibular training to consolidate postural symmetry (29, 30). By heightening body awareness and improving sensorimotor control, this approach can foster more effective postural regulation and help reduce scoliosis-related discomfort (30).

Research has identified AIS neurogenic abnormalities on the convex side of the paraspinal muscles, which may lead to fiber type conversion and grouping (31). This results in bilateral paraspinal muscle imbalance, potentially serving as an underlying driver of scoliosis. Incomplete spinal development, imbalance of muscle strength on both sides of the spine, and greater joint mobility within the spine are all significant causes of scoliosis in adolescents at the peak of skeletal growth (32, 33). Theoretically, Schroth uses isometric measures and various workouts to selectively strengthen the muscles around the scoliotic spine to balance muscle strength between the two sides and to achieve correction. Cervical vertebral alignment and shoulder balance are also significantly improved. The treatment program encompasses three-dimensional postural corrections (active self-correction) to breathing techniques incorporating proprioceptive and exteroceptive stimulation and reflection control (34). This allows for internal expansion of the anterior–posterior diameter of the thoracic cage to reduce vertebral thoracic rotation; external forces applied to the spine promote anterior rotation of the ribs to improve thoracic vertebral rotation (35); and muscle activation stabilizes the expanded rib cage and rotated vertebral position, which may be one reason for its effective improvement in ATR. Exercise can intervene in AIS by correcting neurological abnormalities. The human brain has plasticity, and exercise induces changes in the brain’s central nervous system, generating new neural connections and cells that improve postural control and proprioceptive abnormalities. SEAS therapy emphasizes the patient’s sense of self-correction, which enhances muscle strength and spinal stability. Some studies have shown that SEAS therapy and vestibular rehabilitation exercises significantly improve postural control and balance in patients with AIS. Exercise therapy improves scoliosis by modulating subjective visual, postural, and tactile vertical nerves (36), but more empirical studies are needed to explore its mechanisms.

In summary, Schroth demonstrates the most consistent advantages across all three outcomes—Cobb angle, ATR, and quality of life—and is our overall recommended exercise approach for adolescents with idiopathic scoliosis. While SEAS also provides meaningful benefits, typically smaller than those of Schroth, the limited number of Lyon trials and its modest effect on the Cobb angle indicate that further high-quality, multicenter RCTs are needed to confirm its efficacy. Overall, the balance of structural evidence favors Schroth. However, due to the low quality of evidence, further validation through large-scale, multicenter randomized controlled trials is required to confirm its relative advantages and determine optimal therapeutic doses and monitoring standards. In clinical practice, quality of life is paramount. Exercise intensity and dose should be individualized, considering curve characteristics, functional capacity, and psychological state, and progressed gradually to balance structural gains with quality of life. For adolescents with mild curves, particularly before thoracic deformity develops, management should not restrict school or sports participation, and any activity adjustments should be minimal and symptom-guided.

### 4.1 Study limitations

This review is limited to full-text English-language publications, potentially introducing language bias and excluding relevant evidence published in other languages. Significant heterogeneity exists among the included trials, which may affect pooled effects and lead to inconsistent results. Furthermore, the limited number of trials for Lyon therapy may result in unstable rankings. Collectively, these factors may introduce bias in effect estimates, leading to a lower grade rating. Therefore, caution should be exercised when interpreting the findings. Second, of the 16 studies included in our network meta-analysis, only 7 used a multi-arm design that directly compared the effects of various forms of exercise; thus, many effect size estimates relied on indirect comparisons. To ensure more dependability, it is recommended that future research prioritize conducting more multi-arm design investigations, as they provide more dependable data for direct comparisons compared to indirect comparisons. Although scoliosis-specific exercises have varied names, they share a common purpose. Since Berdishevsky et al. defined the concept of “schools” in 2016 (35), it has become increasingly challenging to distinctly define “Schroth” and other methods within the current landscape as additional “schools” of PSSE have emerged. Therapists are more likely to adopt a blended approach by integrating elements from multiple methods (37). Future studies should aim to identify and define the specific components of each scoliosis intervention method to enhance the treatments’ applicability and the research findings’ reproducibility. For instance, although corrective breathing has been examined (38), its effectiveness still requires further verification, and its dosage and integration across PSSE schools require harmonized +protocols.

Additionally, in this study, although we observed a trend of improvement in the Cobb angle and ATR with different exercise interventions, some of the data did not reach the clinically recognized threshold for treatment effectiveness (39). This suggests that some therapeutic effects are still evident, while the differences between the methods may not have achieved statistical significance or clinically meaningful improvements. However, it is essential to note that the effective therapeutic ranges of the Cobb angle and ATR were 5° and 3°, respectively. If the improvement differences between methods are minor compared to this margin of error, these differences may not be considered clinically significant. Therefore, caution is warranted when interpreting these slight differences. It is recommended that future studies with larger sample sizes and more extended follow-up periods be conducted to further validate whether these trends can translate into significant clinical outcomes.

## 5 Conclusion

This network meta-analysis confirms the positive effects of various exercise therapies in treating AIS, demonstrating significant improvements in the Cobb angle, ATR, and QoL compared to the control group. Schroth therapy was the most effective in correcting the Cobb angle and ATR, followed by SEAS therapy. In contrast, Lyon therapy performed the best in improving quality of life, both of which fall under the category of PSSE. Although exercise interventions were consistently superior to control conditions, the differences among specific exercise modalities were limited in most comparisons. Furthermore, the overall quality of evidence was low, underscoring the need for cautious interpretation. Exercise therapy should be tailored to individual patient characteristics, carefully considering treatment intensity, tolerance, and psychosocial well-being. Given its demonstrated benefits, PSSE deserves greater attention in clinical practice as an adjunctive treatment for AIS. High-quality randomized controlled trials are warranted to clarify the underlying mechanisms further and optimize personalized exercise protocols.

## Data Availability

The raw data supporting the conclusions of this article will be made available by the authors, without undue reservation.
