# Supplementary material for: Effect of exercise therapy on adolescent idiopathic scoliosis in mild to moderate: a systematic review and network meta-analysis
Source: Front Med (Lausanne). 2025 Nov 20;12:1708970. doi: 10.3389/fmed.2025.1708970 (PMC12675335; doi:10.3389/fmed.2025.1708970)
Supplement: Supplementary file 1 [file Data_Sheet_1.docx]

Supplementary Material

Table 1. Characteristics of different intervention methods

Table 2. Inclusion of study risk of bias evaluation results

Table 3. Search strategy

Table 4. Inconsistency of different intervention

Figure 1. Grade level quality assessment

Figure 2. Heterogeneity test (a)cobb, (b)ATR, (c)SRS-22

Figure 3. Sensitivity analysis (a)cobb, (b)ATR, (c)SRS-2

Figure 4. Comparison-adjusted funnel plots of trials included in the network meta-analysis

Figure 5. Begg test

**Table 1 Characteristics of different intervention methods**

| **Physiotherapy School** | **Key Features of Physiotherapeutic Scoliosis-Specific Exercise Approach** |
| --- | --- |
| **Scientific Exercise Approach to Scoliosis (SEAS)** | - Improves patient’s awareness of their deformity to promote self-correction - Autonomous 3D auto-correction - Active stabilization through intensive symmetrical activation of stabilizing muscles - Cognitive-behavioral approach to engage patient and family - Exercises for balance reaction - In-brace PSSE - Improves spinal stability - Home program |
| **Schroth Method** | - Enhances awareness of deformity for self-correction - 3D asymmetric spinal correction exercises (elongation, pelvic alignment, thorax shift, shoulder corrections, derotation with breathing) - Passive mobilization specific to scoliosis - Active 3D stabilization - Corrective breathing - Repetition for body schema correction - Integrates postural corrections in daily activities - Easy and safe home program |
| **The Lyon Approach (LY)** | - Improves postural balance by identifying defects and promoting correction  - Breathing control using expiratory reserve volume with pelvis stabilization  - 3D spine mobilization  - Mobilization of the ilio-lumbar angle (specific to lumbar scoliosis)  - Therapeutic patient education (including diet for cast syndrome prevention and skin care)  - Sitting position check  - Emphasis on endurance of deep paraspinal muscles |
| **Core Stability Exercises** | CS exercise evolves training of the deep trunk muscles to build the balance between internal and external forces, acting over the back and control the trunk in both static and dynamic positions. This approach integrates respiratory control and rib cage position with spinal, scapular, and neck-head positions |
| **Pilates** | It is mainly to exercise the deep small muscle groups of the human body, to maintain and improve the appearance of normal activity posture, to achieve body balance, to create the torso and limbs of the range of motion and ability to move, to emphasise the control of the core muscle groups, to strengthen the human brain on the limbs and the skeletal musculature of the neural sensory and innervation, and then with the correct breathing method of the whole body co-ordination of a movement. |
| **PNF** | It utilises exercise methods that utilise kinaesthetic, postural sensory and other stimuli to enhance relevant neuromuscular responses and promote corresponding muscle contractions.  It is characterised by diagonal and spiral active, passive and resistance movements of the limbs and trunk, and advocates the use of hand contact, verbal commands and visual guidance to influence movement patterns. |

**Table 2 Inclusion of study risk of bias evaluation results**

| study | randomisation | blinding | Assignment hiding | incomplete outcome data | selective reporting | others |
| --- | --- | --- | --- | --- | --- | --- |
| Büyükturan 2024 |  |  |  | Not lost | No | Unclear |
| El 2022 | Computer randomisation |  | Sealed Envelope | Not lost | No | Unclear |
| Khaledi 2022 | Computer randomisation |  |  | Not lost | No | Unclear |
| Ko 2017 |  |  |  | Not lost | No | Unclear |
| Manzak 2023 |  |  |  | Not lost | No | Unclear |
| Kuru 2015 | Computer randomisation |  | Sealed Envelope | Not lost | No | Unclear |
| Monticone 2014 | Random number table | Single-blind |  | Not lost | No | Unclear |
| Kocaman 2021 | Computer randomisation |  |  | Not lost | No | Unclear |
| Hwangbo 2016 |  |  |  | Not lost | No | Unclear |
| MOHAMED 2021 | Computer randomisation |  |  | Not lost | No | Unclear |
| Won 2021 |  | Single-blind |  | Not lost | No | Unclear |
| Kim 2016 |  |  |  | Not lost | No | Unclear |
| Gao 2021 |  |  |  | Not lost | No | Unclear |
| Akyurek 2022 | Computer randomisation |  |  | Not lost | No | Unclear |
| Kisa 2023 | Simple randomization | Single-blind | Sealed Envelope | Not lost | No | Unclear |
| Yuan 2022 |  |  |  | Not lost | No | Unclear |

Note: The table presents the risk of bias assessment for included studies across six domains: randomisation, blinding, assignment hiding, incomplete outcome data, selective reporting, and others. Blank cells indicate that the information was not reported in the study. "Not lost" indicates no missing outcome data, "No" indicates no evidence of selective reporting, and "Unclear" indicates insufficient information to make a judgment. "Sealed Envelope" refers to the method used to conceal allocation.

**Table 3 Search strategy (Take pubmed for example)**

| Search number | Query |
| --- | --- |
| 9 | ((((("Scoliosis"[Mesh]) OR (Idiopathic scoliosis[Title/Abstract])) OR (Adolescent idiopathic scoliosis[Title/Abstract])) OR (Idiopathic scoliosis in adolescents[Title/Abstract]))) AND (((((((((exercise[MeSH]) OR (Exercise interventions[Title/Abstract])) OR (Schroth[Title/Abstract])) OR (SEAS[Title/Abstract])) OR (BAPTS[Title/Abstract])) OR (Core stabilisation exercise[Title/Abstract])) OR (Lyon[Title/Abstract])) OR (Pilates[Title/Abstract])) OR (PNF[Title/Abstract])) |
| 8 | ((((((((exercise[MeSH]) OR (Exercise interventions[Title/Abstract])) OR (Schroth[Title/Abstract])) OR (SEAS[Title/Abstract])) OR (BAPTS[Title/Abstract])) OR (Core stabilisation exercise[Title/Abstract])) OR (Lyon[Title/Abstract])) OR (Pilates[Title/Abstract])) OR (PNF[Title/Abstract]) |
| 7 | Exercise interventions[Title/Abstract] |
| 6 | exercise[MeSH] |
| 5 | ((("Scoliosis"[Mesh]) OR (Idiopathic scoliosis[Title/Abstract])) OR (Adolescent idiopathic scoliosis[Title/Abstract])) OR (Idiopathic scoliosis in adolescents[Title/Abstract]) |
| 4 | Idiopathic scoliosis in adolescents[Title/Abstract] |
| 3 | Adolescent idiopathic scoliosis[Title/Abstract] |
| 2 | Idiopathic scoliosis[Title/Abstract] |
| 1 | "Scoliosis"[Mesh] |

**Table 4 Global inconsistency of different intervention**

| Parameters | chi2 | P value |
| --- | --- | --- |
| Cobb | 7.77 | 0.1005 |
| SRS-22 | 1.90 | 0.1686 |

Note: Global inconsistency test results for the network meta-analysis. Non-significant p-values indicate acceptable consistency between direct and indirect comparisons.

**Figure 1 Grade level quality assessment**


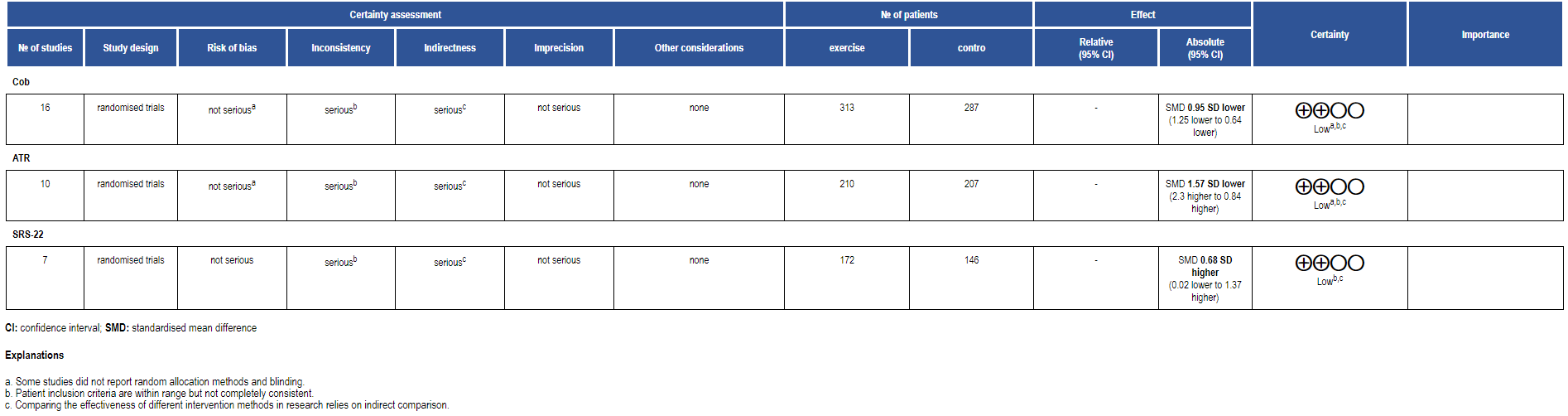


**Figure 2 Heterogeneity test (a)cobb, (b)ATR, (c)SRS-22**

(a)cobb

-
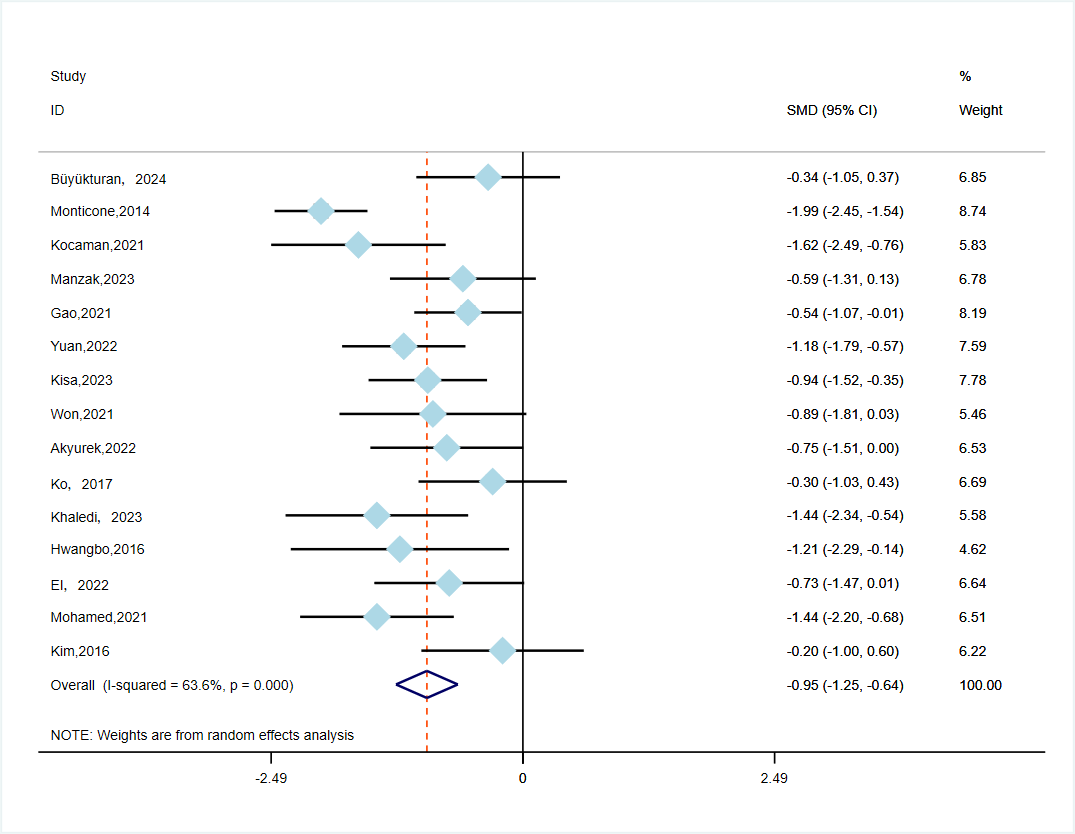

- (b) ATR
-
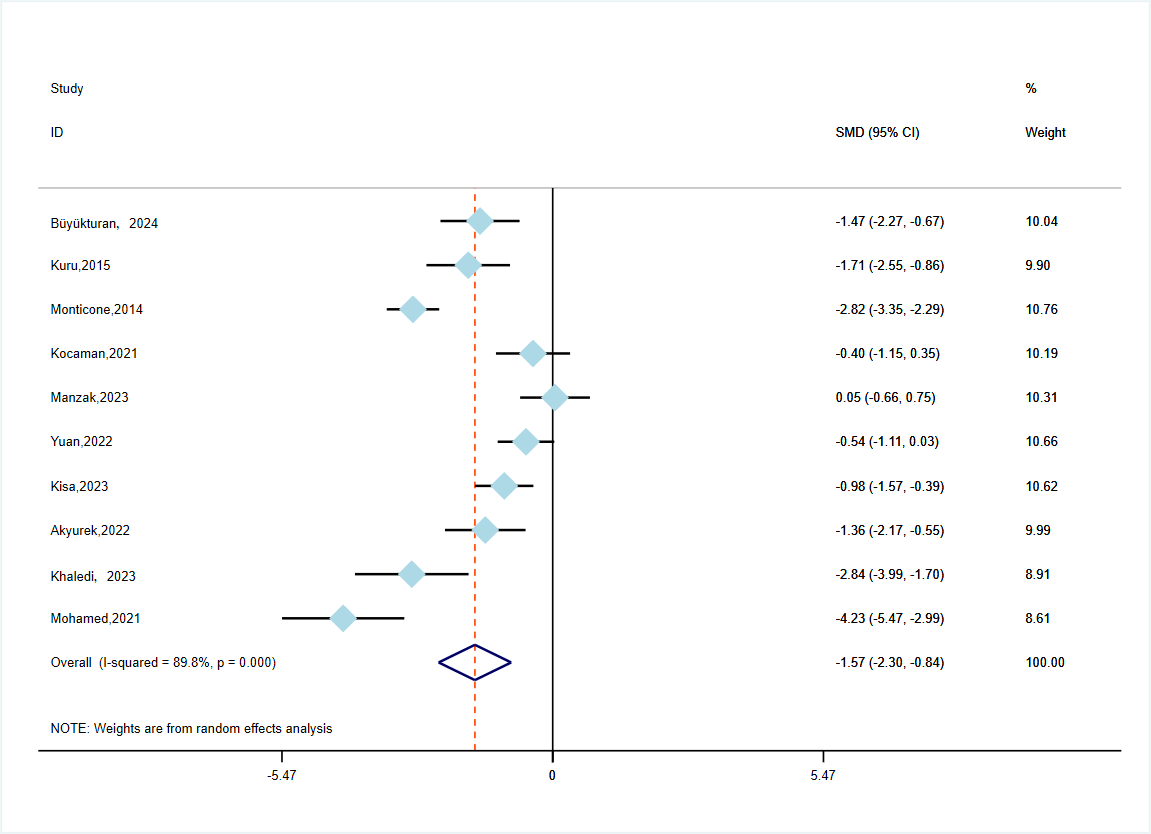


(c)SRS-22


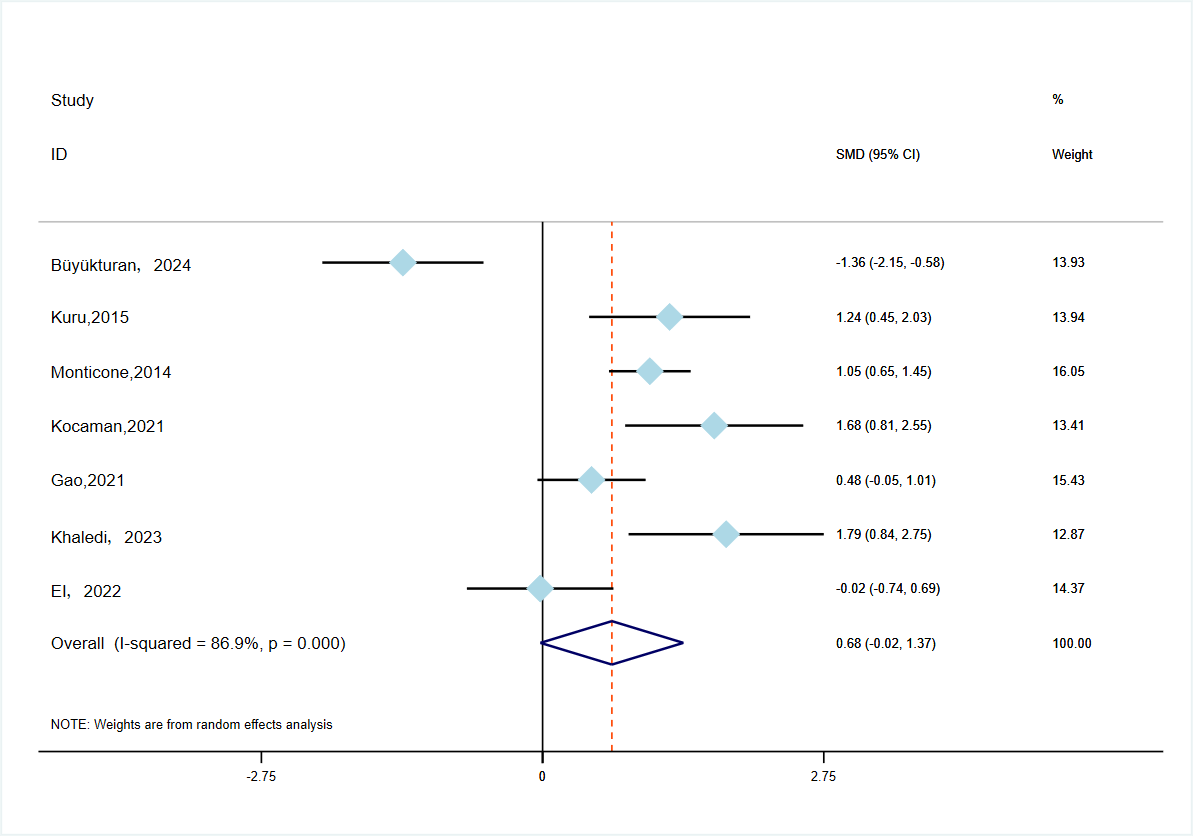


Note: I^2^ less than 50 is low heterogeneity, 50-70 is medium heterogeneity, and greater than 70 is high heterogeneity

**Figure 3 Sensitivity analysis. (a)Cobb, (b)ATR, (c)SRS-22**


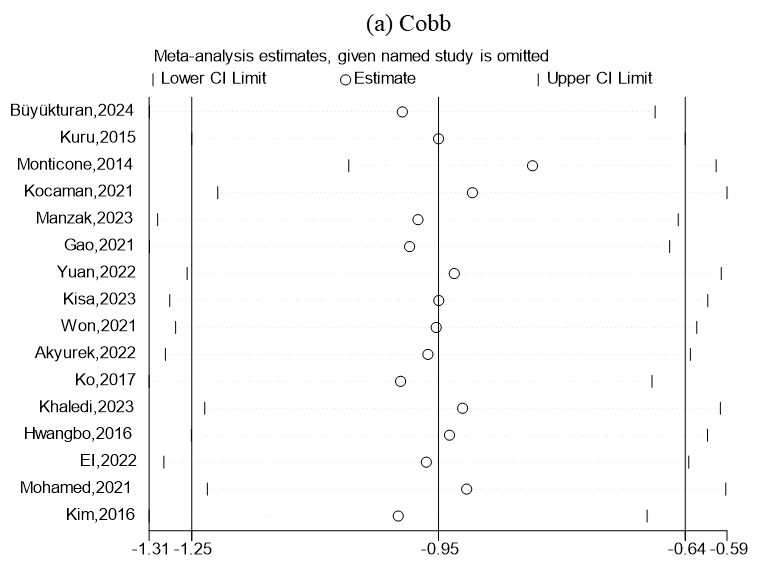

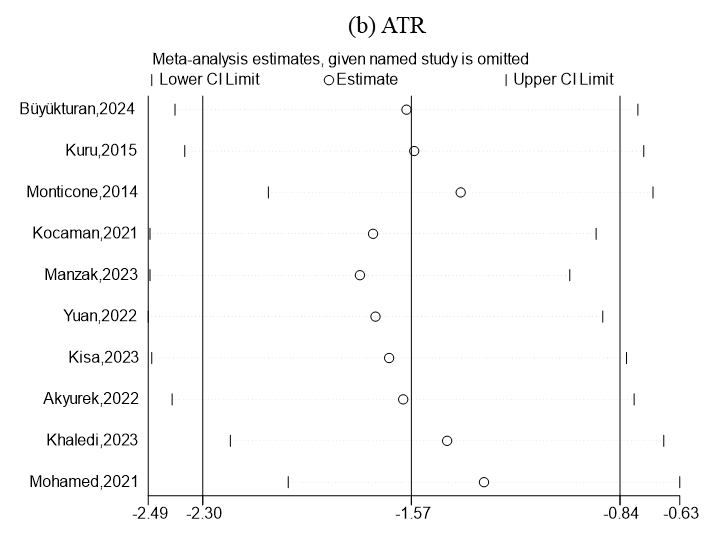


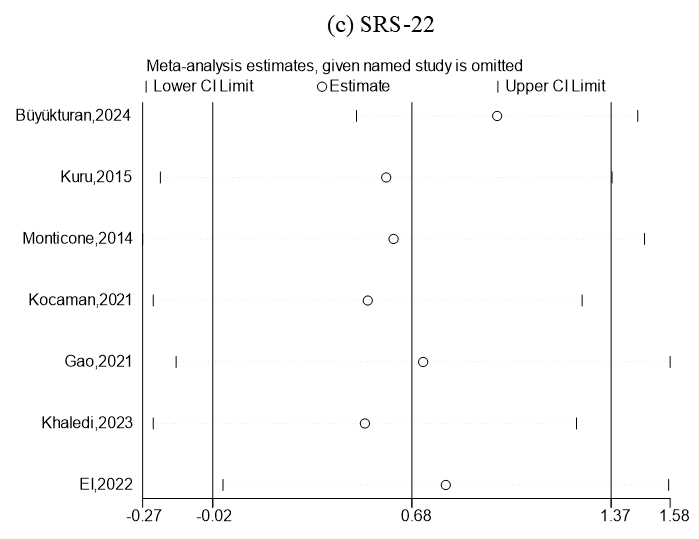


**Figure 4 Funnel plot**

**
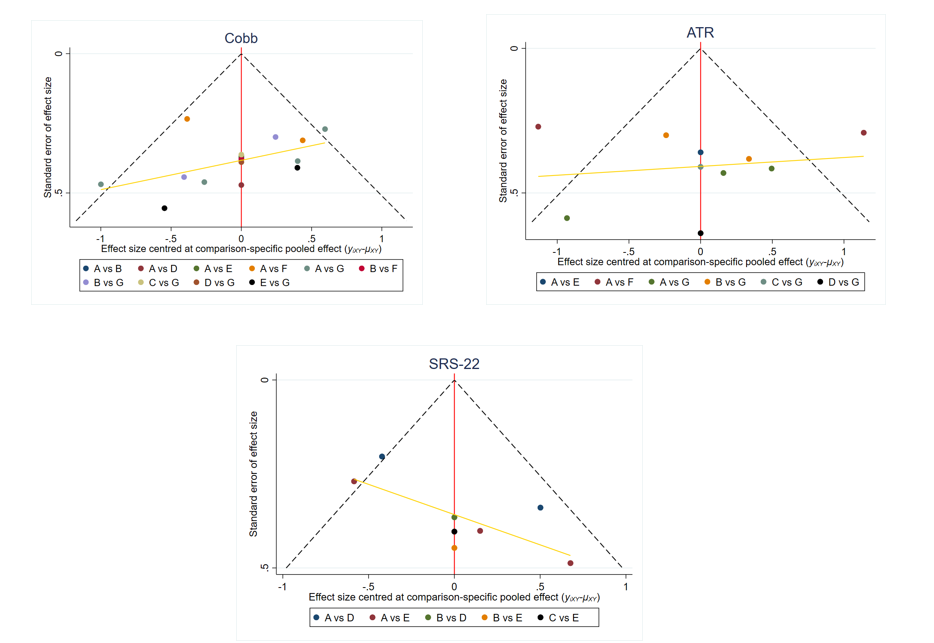
**

Note: The funnel plot shows the distribution of standard errors of effect sizes between different comparison groups. Each point in the plot represents a different comparison group (e.g., A vs. B, B vs. F, etc.). The horizontal axis represents the effect values centred on the specific pooled effect size of the comparison group, and the vertical axis represents the standard error.

**Figure 5 Begg test**

**
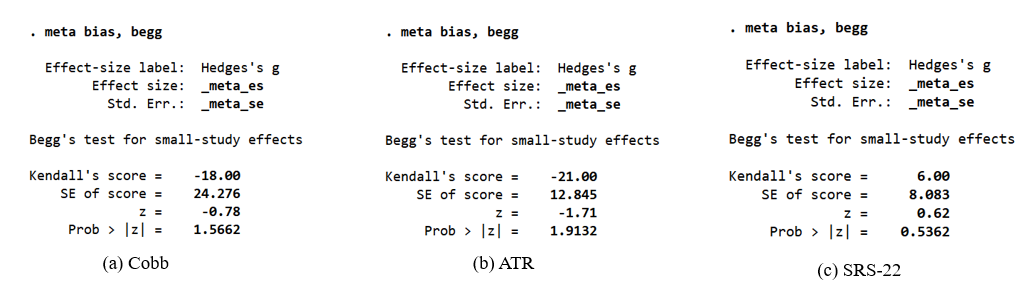
**

Note: Begg's test is used to assess the impact of small-study effects on meta-analysis results. The figure shows the test results for three different datasets (Cobb, ATR, SRS-22), including Kendall's scores, standard errors (SE), z-statistics, and two-sided test p-values (Prob > |z|). Effect sizes are uniformly expressed using Hedges's g, with their values (_meta_es) and standard errors (_meta_se) listed above each group's test results. (a) Cobb: The results did not show significant small-study effects (z = -0.78, p = 1.5662). (b) ATR: The results did not show a significant small-sample effect (z = -1.71, p = 1.9132). (c) SRS-22: The results did not show a significant small-sample effect (z = 0.62, p = 0.5362).
